# A radiographic and physical analysis of factors affecting seat belt position in sitting car seat

**DOI:** 10.1038/s41598-022-14607-x

**Published:** 2022-06-24

**Authors:** Hiroki Yamagata, Norihiro Nishida, Tomohiro Izumiyama, Ryusuke Asahi, Masahiro Koike, Atsushi Mihara, Yasuaki Imajo, Hidenori Suzuki, Masahiro Funaba, Shigeru Sugimoto, Masanobu Fukushima, Takashi Sakai

**Affiliations:** 1 Department of Orthopedic Surgery, Yamaguchi University Graduate School of Medicine, 1-1-1 Minami-Kogushi, Ube City, Yamaguchi 755-8505 Japan; 2 Vehicle Development Division, Crash Safety Development Department, Mazda Motor Corporation, 3-1, Shinchi, Fuchu-cho, Aki-gun, Hiroshima 730-8670 Japan; 3 Department of Radiological Technology, Yamaguchi University Hospital, 1-1-1 Minami-Kogushi, Ube City, Yamaguchi 755-8505 Japan

**Keywords:** Orthopaedics, Biomechanics, Trauma

## Abstract

The characteristic subcutaneous hemorrhage along the seat belt in motor vehicle accidents is called the seat belt sign (SBS). The risk of organ injuries is especially high when abdominal SBS is located above the anterior superior iliac spine (ASIS). The purpose of this study analyzed the physical and radiographic factors of healthy volunteers sit on car seat that affect initial position of abdominal seat belt, namely “lap belt”, related to the seat belt injury. This study was examined prospectively relation between physical characteristics of one hundred healthy volunteers and lap belt position sitting the car seat. Physical findings were clarified age, sex, height, body mass index (BMI), and waist circumference. Radiographical findings were measured lumber lordosis (LL), sacral slope (SS), and initial lap belt position by marking with lead tape for the center and ASIS of the lap belt installed on the driver’s car seat. In the lateral X-ray image, we measured the horizontal distance (X-value) and vertical distance (Z-value) from the ASIS to the central marker. The lap belt angle was determined to measure the angle between the horizontal line and the straight line connecting the upper edges of the markers. Statistical analysis of the relationships between physical characteristics and radiological findings was performed. X-value and Z-value were positively correlated with body weight, BMI, and waist circumference, while the lap belt angle was negatively correlated with body weight, BMI, and waist circumference. The relationship between physical characteristics and the initial position of seat belt was analyzed. Since the lap belt is positioned higher than the ASIS in occupants with a high BMI, it is likely to cause seat belt injury. This analysis can help to develop safer seat belts and to enlighten car occupants.

## Introduction

In the early 1900s, as motorization in the United States led to automobile use becoming widespread among the public, the number of traffic accidents increased dramatically. According to the Metropolitan Life Insurance Company Statistical Bulletin in 1922, the number of automobile fatalities in 50 cities in the United States was 3637 (149.7 per 1 million population) in 1920 and 3837 (155.1 per 1 million population) in 1921, and it was reported that traffic accidents as a cause of death had come to the fore ^1^. After that, the seat belts used in aircraft etc. were adopted for automobiles and adapted improved for safety in the event of a traffic accident. Since the prototype of the current three-point seat belt in 1959, its use has spread throughout the world.

Seatbelts have helped to significantly reduce occupant mortality ^2,3^, but as seatbelts have become more commonplace, injuries caused by seatbelts during traffic accidents have increased ^4,5^. In 1962 Garrett et al. first reported a seatbelt injury, calling it “the seatbelt syndrome” ^6^. Doersch and Dozier named the characteristic subcutaneous hemorrhagic spots on the chest and abdomen that correspond to the shape of the seat belt in traffic accidents the seat belt sign (SBS) ^7^. Jiang et al. reported a higher risk of seatbelt injury, especially if the abdominal SBS was located above the anterior superior iliac spine (ASIS) ^8^. The mechanism of why the lap belt (the abdominal part of the seatbelt) does not catch on the iliac crest but causes abdominal damage above the ASIS is still unknown. In the laboratory, it is necessary to separate the lap belt position into static and dynamic factors. The position of the SBS is the sum of the lap belt position during driving (initial position) and the amount of shift during a collision (upward shift). We hypothesized that the initial position of the same seat belt may change due to individual differences in age, sex, body shape and spinopelvic alignment, which would affect the position of the SBS. The aim of this study was to investigate the physical parameters that affect the initial position of the lap belt related to seat belt injury, and to contribute to the development of a safer car seat and belt.

## Materials and methods

**Figure 1:**
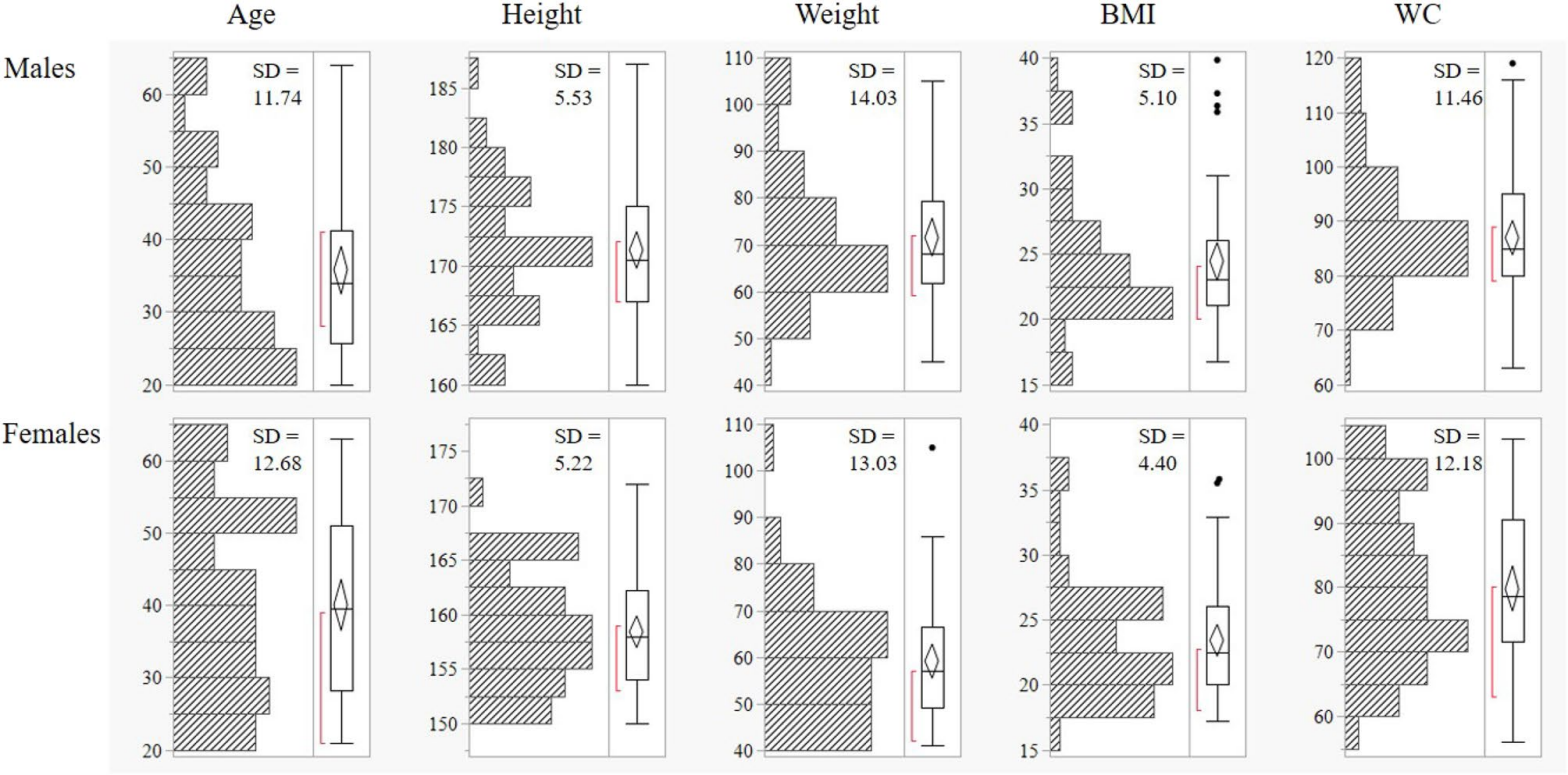
Histograms and standard deviations (SD) for age, height, weight, BMI, and WC are shown.

This study was conducted in collaboration with project partner Mazda Motor Corporation (Aki-gun, Hiroshima, Japan), with approval from the ethical review board of the corresponding author’s host institute. Our study provided no information that is beneficial to the industry collaborator who provided the car seat that we used. And the individual (for example, Fig. 1b) in this manuscript has given written informed consent to publish these case details. The present study was approved by the Ethics Committee at the Center for Clinical Research, Yamaguchi University Hospital (Ube, Japan; approval no. H2019-013). All methods were performed in accordance with the relevant guidelines and regulations.

The subjects were 100 healthy volunteers (50 males and 50 females) with no history of spinal or lower limb disease or surgery (Table 1, Fig. 1). The mean age was 37.9 years (20–64 years), the mean height was 164.9 cm (150–187 cm), the mean weight was 65.3 kg (42–105 kg), the mean Body Mass Index (BMI) ^9^ was 23.9 kg/m^2^ (16.7–39.8 kg/m^2^), and the mean waist circumference (WC) was 83.4 cm (56–119 cm). WC was measured at the umbilical level, which is defined by the Japan Society for the Study of Obesity and is the standard method for diagnosing metabolic syndrome in Japan ^10^.

**Table 1:** Participants’ characteristics.

Parameter	Males	Females	*p* value
*n*	50	50	
Age (y.o.)	35.8 (20–64)	40.0 (21–63)	0.08
Height (cm)	171.3 (160–187)	158.4 (150–172)	< 0.01
Weight (kg)	71.6 (45–105)	59.0 (42–105)	< 0.01
Body mass index (kg/m^2^)	24.4 (16.7–39.8)	23.4 (17.3–35.8)	0.31
Waist circumference (cm)	87.0 (75–119)	79.8 (56–103)	< 0.01
			Mann–Whitney *U* test

**Figure 2:**
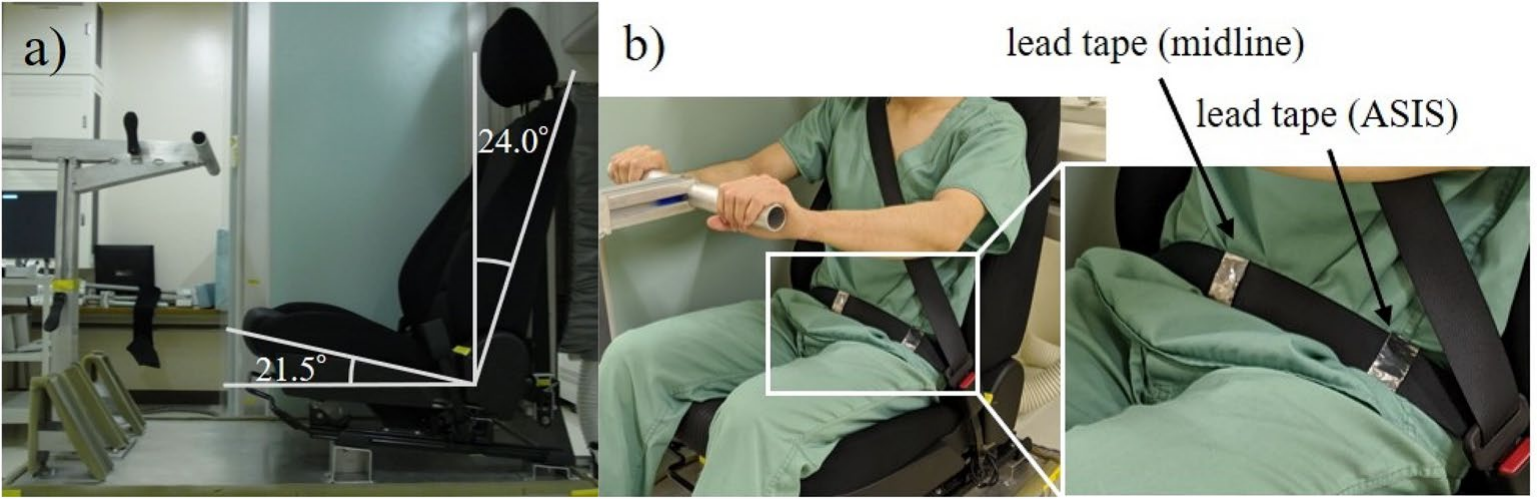
X-ray table using Mazda 3 car seat. (**a**) The back of the seat was fixed at 24 degrees, and the seat surface at 21.5 degrees. The handle that imitates the steering wheel, and the pedals, were placed in the same positions as in the actual vehicle. (**b**) The midline and left ASIS equivalent positions of the lap belt were marked by wrapping 3 M™ lead foil tape 420 (25.4 mm × 32.9 mm) around each position so that they could be identified radiographically. In each case, the first author palpated and confirmed the center of the abdomen and the left ASIS, and positioned the markings.

Using the seat on the driver’s side of the Mazda 3 vehicle, we created a radiography table designed to fit within the X-ray imaging range. The back of the seat was fixed at 24 degrees, and the seat surface at 21.5 degrees. The handle that imitates the steering wheel, and the pedals, were placed in the same positions as in the actual vehicle. The front-rear direction of the handle could be adjusted to the position that the subject felt most natural. The lap belt was marked with 3 M™ lead foil tape 420 so that the midline position and the position corresponding to the left ASIS could be distinguished on radiographs (Fig. 2).

**Figure 3:**
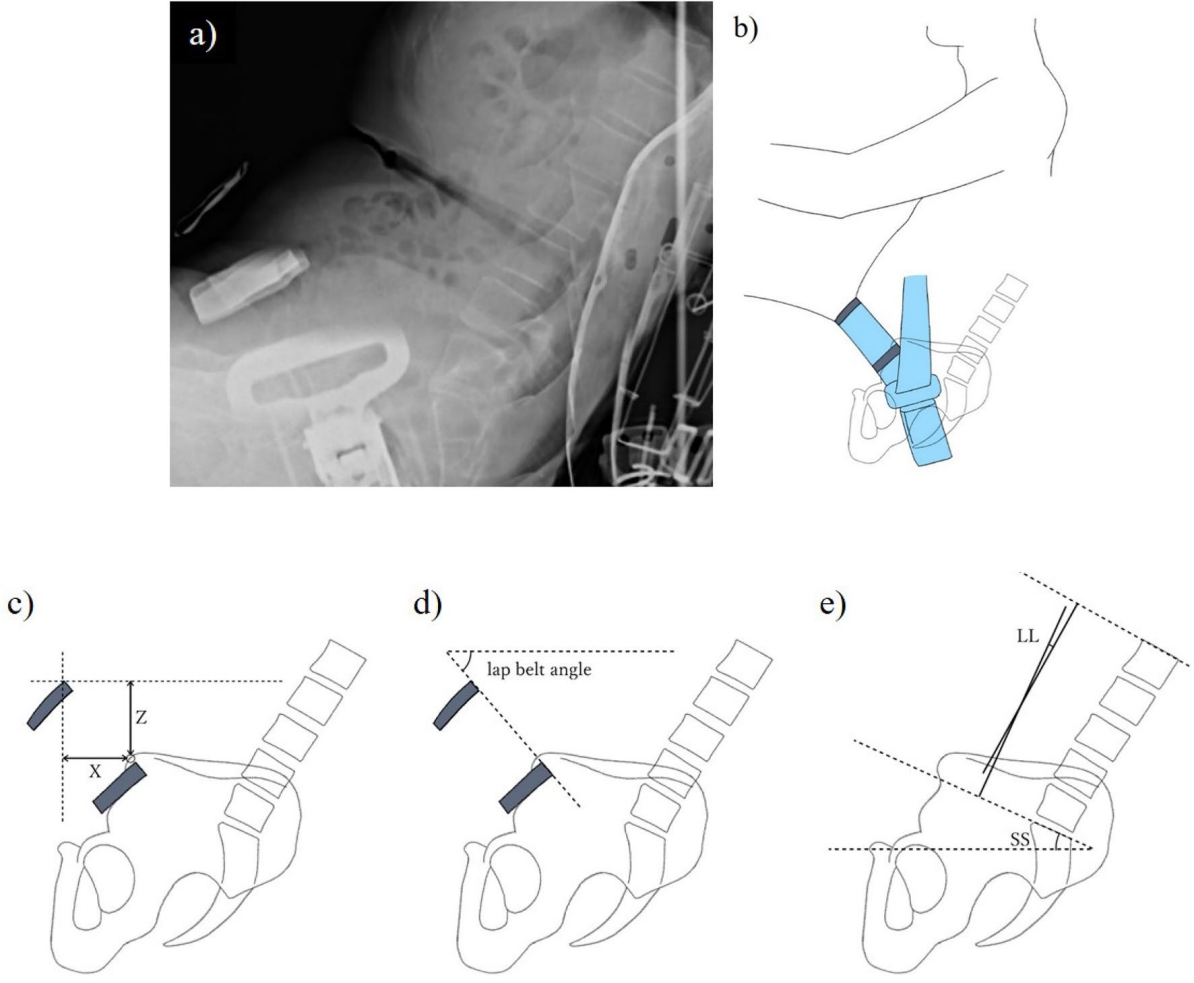
Seated lumbopelvic lateral radiograph (**a**) and illustration (**b**). The horizontal distance between the vertical line drawn from the ASIS and the upper edge of the midline marker was measured as the X-value, and the vertical distance between the horizontal line drawn from the ASIS and the upper edge of the midline marker was measured as the Z-value (**c**). The lap belt angle was measured as the angle between the straight line connecting the upper edge of the markers and the horizontal line (**d**). Lumbar lordosis (LL) was defined as the angle between the first lumbar superior endplate and the first sacral superior endplate, and sacral slope (SS) was defined as the angle between the first sacral superior endplate and the horizontal line, were measured for the lumbopelvic alignment (**e**).

Next, a seated lateral X-ray of the pelvis from the lumbar spine of the subject seated on the radiography table was performed. In order to minimize X-ray exposure, the seat angle was fixed, and only one image was taken. The positional relationships between the ASIS and the midline marker, the lap belt angle, and lumbopelvic alignment were measured using the X-ray images. The positional relationship between the ASIS and the midline marker was measured using the horizontal distance between the vertical line drawn from the ASIS and the upper edge of the midline marker as the X-value, and the vertical distance between the horizontal line drawn from the ASIS and the upper edge of the midline marker as the Z-value. The lap belt angle was measured as the angle between the straight line connecting the upper edge of the markers and the horizontal line. Lumbar lordosis (LL) as the angle between the first lumbar superior endplate and the first sacral superior endplate, and sacral slope (SS) as the angle between the first sacral superior endplate and the horizontal line, were measured for lumbopelvic alignment (Fig. 3) ^11–13^.

**Figure 4:**
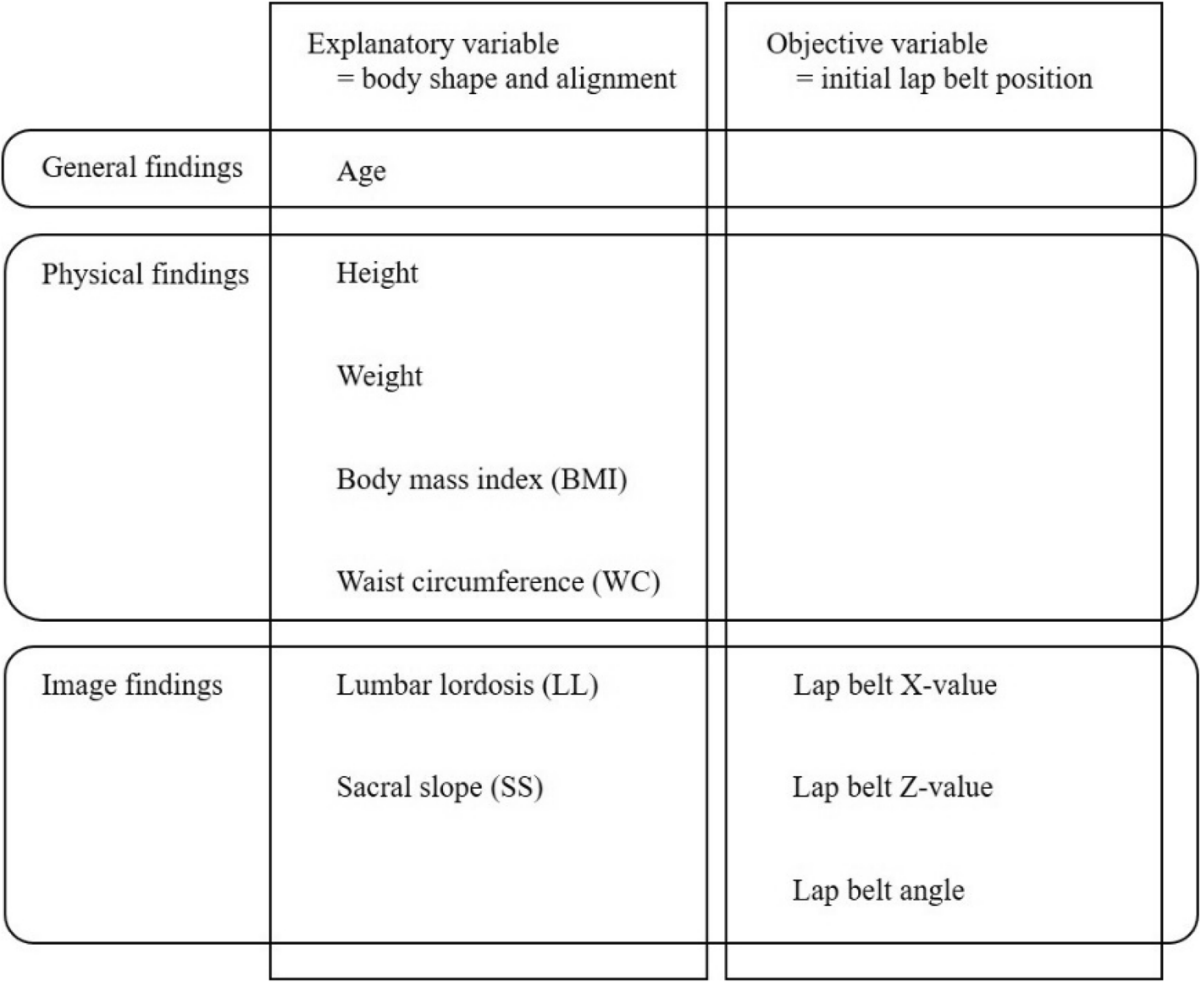
Explanatory variables and objective variables. Age and physical characteristics of height, weight, Body mass index (BMI) ^10^, and Waist circumference (WC) were used as explanatory variables. Among the X-ray findings, Lumbar lordosis (LL) and Sacral slope (SS) were used as explanatory variables, while the X-value of the lap belt, the Z-value of the lap belt, and lap belt angle were used as objective variables.

The relationships between the unique body shape parameters and the lap belt parameters were examined. The objective variables were the lap belt X-value, lap belt Z-value, and lap belt angle, indicating the initial lap belt position, and the explanatory variables were age, height, weight, BMI, WC, LL, and SS (Fig. 4). Spearman’s rank correlation coefficient was used to examine the correlations. The stepwise method was used for multiple regression analysis. Analysis was performed using StatFlex (version 6; Artech Co., Ltd. Osaka, Japan), and *p* values < 0.05 were considered significant.

## Results

**Figure 5:**
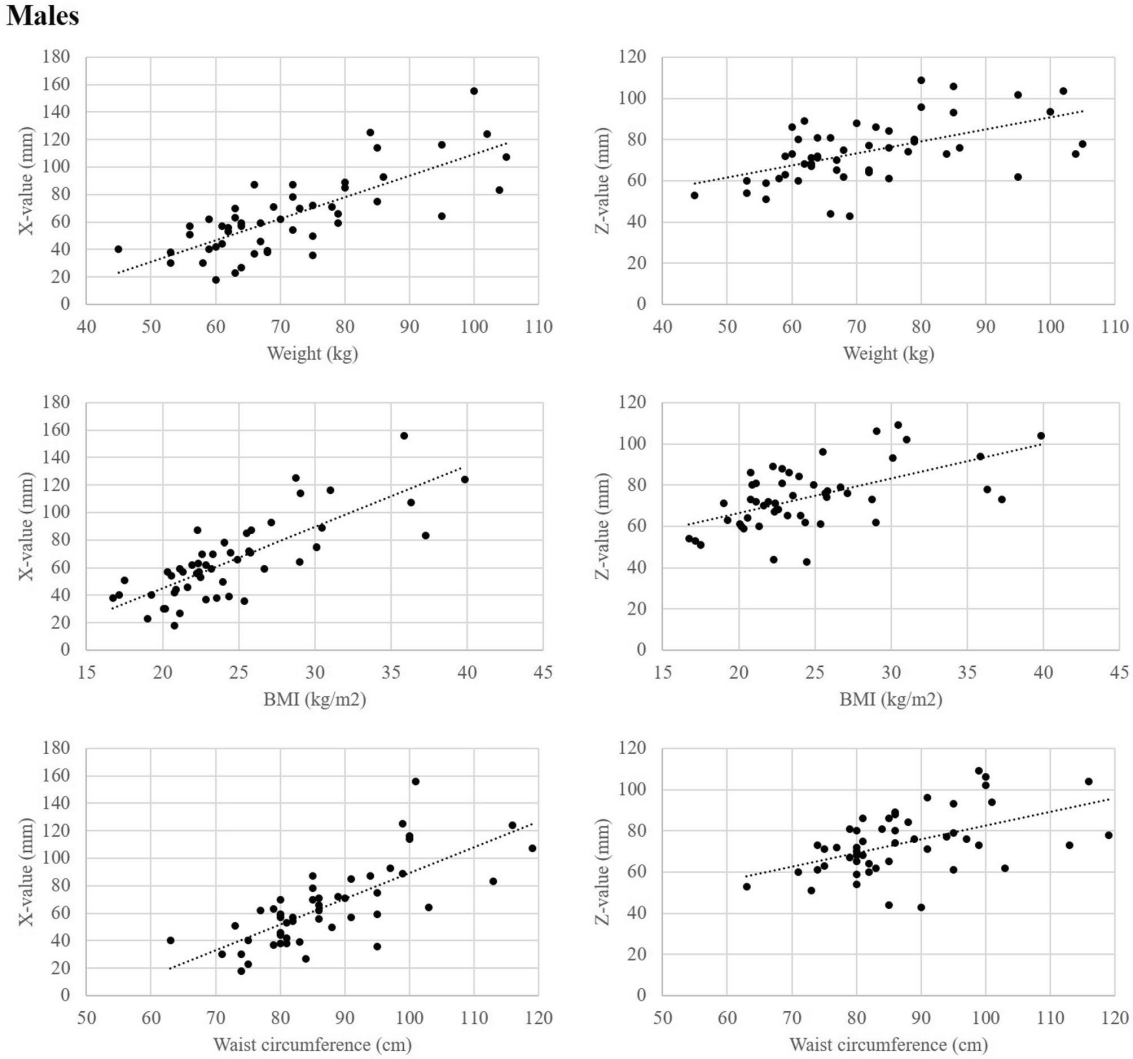

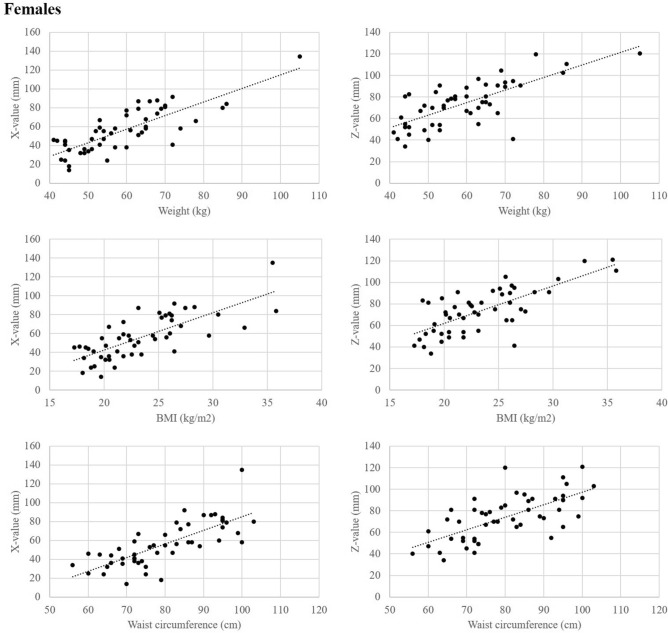
Relationships between weight, BMI, or waist circumference and X-value or Z-value.

There were significant differences between males and females in height (171.3 cm vs. 158.4 cm) and weight (71.6 kg vs. 59.0 kg). The analyses were performed by sex, considering the sex differences in body shape. In males, X-value was positively correlated with age (*r* = 0.39), weight (*r* = 0.73), BMI (*r* = 0.77), and WC (*r* = 0.74). Z-value was positively correlated with weight (*r* = 0.56), BMI (*r* = 0.56), and WC (*r* = 0.52). The lap belt angle was negatively correlated with weight (*r* =  − 0.33), BMI (*r* =  − 0.35), and WC (*r* =  − 0.37). Height, LL, and SS were not correlated with X-value, Z-value, or lap belt angle. In females, X-value was positively correlated with age (*r* = 0.30), height (*r* = 0.40), weight (*r* = 0.77), BMI (*r* = 0.75), and WC (*r* = 0.74). Z-value was positively correlated with height (*r* = 0.31), weight (*r* = 0.67), BMI (*r* = 0.64), and WC (*r* = 0.69). The lap belt angle was not correlated with any other parameters. As with males, LL and SS did not correlate with X-value, Z-value, or lap belt angle (Table 2). In both males and females, weight, BMI, and WC were strongly correlated with X-value and Z-value (Fig. 5). Multiple regression analysis was conducted for each sex. The results of multiple regression analysis using the stepwise method showed that BMI was the factor most strongly correlated with both X-value and Z-value. In males, lap belt angle was most strongly correlated with BMI (Table 3).

**Table 2:** Correlation between each explanatory variable and the X-value, Z-value, and lap belt angle by sex.

Parameter	*r*			*p*		
	X	Z	Angle	X	Z	Angle
**Males (*n* = 50)**						
Age	0.391	− 0.012	− 0.210	0.0050	0.9358	0.1443
Height	− 0.096	− 0.001	0.242	0.5090	0.9932	0.0903
Weight	**0.729**	**0.562**	− 0.330	< 0.0001	< 0.0001	0.0191
BMI	**0.773**	**0.555**	− 0.351	< 0.0001	< 0.0001	0.0122
WC	**0.741**	**0.522**	− 0.373	< 0.0001	0.0001	0.0076
LL	0.003	− 0.202	− 0.162	0.9848	0.1587	0.2623
SS	0.070	− 0.147	− 0.039	0.6296	0.3093	0.7888
**Female (*n* = 50)**						
Age	0.302	0.132	− 0.119	0.0331	0.3601	0.4122
Height	0.405	0.311	− 0.183	0.0035	0.0280	0.2036
Weight	**0.765**	**0.669**	− 0.215	< 0.0001	< 0.0001	0.1335
BMI	**0.748**	**0.640**	− 0.218	< 0.0001	< 0.0001	0.1409
WC	**0.788**	**0.686**	− 0.164	< 0.0001	< 0.0001	0.2565
LL	− 0.017	− 0.021	− 0.028	0.9057	0.8871	0.8449
SS	− 0.027	0.003	− 0.032	0.8524	0.9865	0.8276

Correlation coefficient (*r*) and *p* values are shown.

**Table 3:** Multiple regression analysis using the stepwise method are shown (β: partial regression coefficient, *SE*(β): standard error of β, stdβ: standardized partial regression coefficient).

Objective variable: X-value, male (*n* = 50)						
Degree	Variable	β	*SE*(β)	stdβ	*t*	*p*
0		− 32.45	13.30			
1	**BMI**	**4.530**	**0.4912**	**0.7987**	**9.222**	** < 0.0001**
2	LL	− 0.7501	0.4089	− 0.2467	− 1.834	0.0731
3	SS	1.210	0.5144	0.3153	2.353	0.0230
Multiple correlation coefficient *R* = 0.812						
Coefficient of determination *R*^2^ = 0.659						
Adjusted *R*^2^ = 0.637						
RMSE = 17.4						

Objective variable: Z-value, male (*n* = 50)						
Degree	Variable	β	*SE*(β)	stdβ	*t*	*p*
0		31.17	8.756			
1	**BMI**	**1.727**	**0.3494**	**0.5810**	**4.943**	** < 0.0001**
2	LL	− 0.3126	0.1874	− 0.1961	− 1.668	0.1019
Multiple correlation coefficient *R* = 0.597						
Coefficient of determination *R*^2^ = 0.356						
Adjusted *R*^2^ = 0.328						
RMSE = 12.4						

Objective variable: lap belt angle, male (*n* = 50)						
Degree	Variable	β	*SE*(β)	stdβ	*t*	*p*
0		91.21	8.678			
1	**BMI**	− **1.602**	**0.3464**	− **0.5473**	− **4.625**	** < 0.0001**
2	LL	− 0.2747	0.1857	− 0.1750	− 1.479	0.1458
Multiple correlation coefficient *R* = 0.589						
Coefficient of determination *R*^2^ = 0.347						
Adjusted *R*^2^ = 0.319						
RMSE = 12.3						

Objective variable: X-value, female (*n* = 50)						
Degree	Variable	β	*SE*(β)	stdβ	*t*	*p*
0		− 59.83	12.36			
1	Age	0.5587	0.1561	0.3033	3.579	0.0008
2	**BMI**	**3.984**	**0.4465**	**0.7510**	**8.922**	** < 0.0001**
3	LL	0.2903	0.2159	0.1139	1.344	0.1854
Multiple correlation coefficient *R* = 0.821						
Coefficient of determination *R*^2^ = 0.674						
Adjusted *R*^2^ = 0.653						
RMSE = 13.8						

Objective variable: Z-value, female (*n* = 50)						
Degree	Variable	β	*SE*(β)	stdβ	*t*	*p*
0		− 16.41	12.98			
1	Age	0.2137	0.1630	0.1287	1.311	0.1964
2	**BMI**	**3.487**	**0.4695**	**0.7292**	**7.427**	** < 0.0001**
Multiple correlation coefficient *R* = 0.740						
Coefficient of determination *R*^2^ = 0.547						
Adjusted *R*^2^ = 0.528						
RMSE = 14.5						

Objective variable: lap belt angle, female (*n* = 50)						
Degree	Variable	β	*SE*(β)	stdβ	*t*	*p*
0		88.72	11.97			
1	Age	− 0.2441	0.1503	− 0.2232	− 1.624	0.1110
2	BMI	− 0.7937	0.4327	− 0.2521	− 1.834	0.0730
Multiple correlation coefficient *R* = 0.335						
Coefficient of determination *R*^2^ = 0.113						
Adjusted *R*^2^ = 0.075						
RMSE = 13.3						

Significant values are in bold.

## Discussion

It has been reported that the risk of seat belt injury is higher in cases where the SBS is located higher-up ^8^. We analyzed differences in personal body shape parameters affect the position of the SBS and investigated relationship between body shape and the initial lap belt position when sitting in a car seat.

The prototype of the three-point seatbelt was first installed in production vehicles in 1959, and its basic structure has remained unchanged for over 60 years. It is specified as standard equipment in the seat belt laws of many countries, and most of the major risks associated with traffic accidents are lower for seat belt users ^14^. However, as seat belts have become more widely used, there have been more reports of belt-induced injuries. Garrett and Braunstein were the first to report various organ injuries associated with seat belts, and called it seat belt syndrome ^6^. Chance fracture, a flexion distraction fracture of the thoracolumbar spine, is famous for its high complication rate in traffic accidents due to the strong flexion force at the lap belt, and is now also called “seat belt” fracture ^15,16^. When treating patients with seat belt syndrome, it is important to be aware of complications such as intestinal and mesenteric injuries in the intra-abdominal organs ^4^ and arterial injuries in the retroperitoneal organs ^5^. Doersch and Dozier described a characteristic compression scar as a sign to suspect seat belt syndrome, and called it the “SBS” ^7^. SBS refers to subcutaneous hemorrhagic spots consistent with the belt site and is associated with intra-abdominal organ damage ^17^, although there may be no associated injury even when SBS is present ^18^. Johnson and Eastridge reported that if the SBS is superior to the ASIS, there is an increased risk of intra-abdominal injury and abdominal surgery, and if the SBS is inferior to the ASIS, there is an increased risk of pelvic fracture, which is an indication and predictor of abdominal surgery ^19^. Assuming that the same energy is applied, the abdominal organs are more likely to be damaged than the pelvis, and therefore the presence of SBS above ASIS is considered to be a risk for seat belt syndrome. However, the mechanism by which bleeding spots caused by seat belts, which should be on the ASIS, occur above the ASIS is still unclear, and therefore no preventive measures have been established.

We assumed that the position of the lap belt, related to seatbelt injury at the time of the crash, can be divided into a static factor, the initial lap belt position to sit on car seat, and a dynamic factor, for example upward shift of seatbelt and rotation of pelvis. And SBS can be determined by adding these two factors. This study identified physical characteristics that affect the initial lap belt position. There were few reports that analyzed physical characteristics were related to injuries in car accidents.

First, it has been reported that higher BMI is associated with higher risk of damage and mortality in occupants ^20–22^. Zarzaur et al. hypothesized that this was due to inappropriate use of seat belts and investigated the effects of seat belts and obesity on the presence of intra-abdominal injuries ^23^. They found that intra-abdominal organ injury and mortality were not associated with seat belt use in obese occupants, but the risk of abdominal injury with AIS 1 or higher was. With regard to mortality, Elkbuli et al. also reported that seat belt use reduces mortality regardless of BMI ^24^, so there is no disagreement that seat belt use is desirable even in obese occupants. However, these reports focused only on obesity and did not take into account age, sex, and height as in the present study.

About SBS location, Hartka et al. measured the position of the SBS in actual car accident patients on CT, and reported that the X-value was greater in obese people while the Z-value did not change much ^25^. In contrast, at the laboratory level, Reed et al. measured the lap belt position, and reported that a 10 kg/cm^2^ increase in BMI resulted in a 43 mm anterior and 21 mm superior position ^26^. They also mentioned that the distance from the pelvis, and thus the possibility that the belt will not properly rest on the pelvis, will increase.

**Figure 6:**
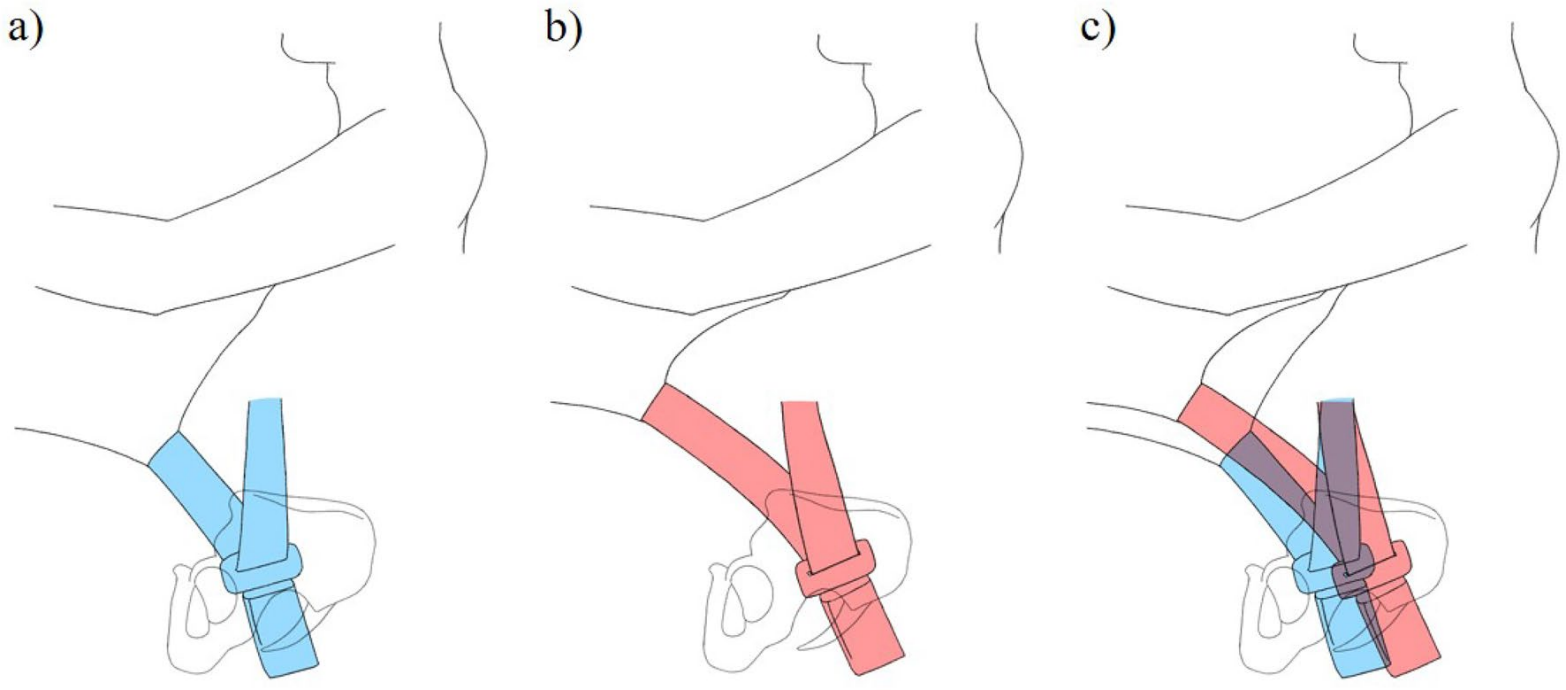
The relationship between the pelvis and seat belt position of a lower BMI occupant (**a**) and the pelvis and seat belt position of a higher BMI occupant (**b**) are shown. In comparison, the X and Z values are larger, and the lap belt angle is smaller for the higher BMI occupant (**c**). This may facilitate the submarine phenomenon where the pelvis goes under the lap belt.

In this study, weight, BMI, and WC all influenced the X-value, Z-value, and lap belt angle. These variables are all factors associated with obesity. BMI was found to be most strongly correlated with X-value and Z-value, and the higher the BMI, the higher the Z-value. In males, BMI was most strongly correlated with lap belt angle, and the higher the BMI, the smaller the lap-belt angle. A small lap belt angle may make it easier for the pelvis to submarine, increasing the upward shift in an accident. Obese people may therefore have a higher risk of seatbelt injury due to higher SBS caused by higher Z-value and larger upward shift (Fig. 6).

The reason for the difference between our results and Hartka et al. ^25^ may be the difference in posture when measuring the X-value and Z-value. Their CT study would have been measured in the supine position, but our study used a coordinate system in the sitting position. It is known that the total spinal alignment in the seat sitting position is significantly different from that in the supine position, especially the posterior tilt of the pelvis ^27–29^. Therefore, there may be differences in the X- and Z-values calculated from CT taken in the supine position.

Second, height had no effect on the initial lap belt position in most cases. Only the X- and Z-values in females were significantly correlated with height. There are no reports that mention height and SBS, nor are there any reports that height is related to spinopelvic alignment in the sitting position. Compared to the results for weight and BMI mentioned above, the relevance is extremely low, so we consider height to be negligible. However, since women were on average about 12 cm shorter than men in this study, it is possible that height and initial position may be correlated only in the height range below a certain level.

Third, LL and SS were not correlated with X-value, Z-value, or lap belt angle. There have been no reports mentioning the relationship between spinopelvic alignment and SBS. Buckland et al. reported that the pelvis tends to tilt posteriorly in obese individuals ^30^. Nishida et al. investigated changes in spinal alignment in the sitting position while seated in a car seat, and reported that there were differences depending on body shape even in the same seat ^31^. In this study, there were also no significant correlations between weight or BMI and LL or SS. Therefore, lumbar pelvic alignment is not considered to be involved in the change of initial lap belt position in obese subjects. However, lumbopelvic alignment is related to sitting posture, and sitting in an inappropriate posture may be associated with seat belt injury. Richardson et al. conducted crash tests using a cadaver on a car seat that was reclined 50 degrees, and reported several damage patterns ^32^. Further research is needed on the relationship between sitting posture and traffic trauma including seat belt injury.

### Limitations

The first limitation was that the X-ray images were taken immediately after sitting. The sitting posture may change over time due to differences in muscle fatigue or comfort. Therefore, no matter when an image was taken, it could not be considered a permanent alignment.

Secondly, the radiography was performed at a single, predetermined seat angle. A different angle or height of the seat or height of the steering wheel may have led to different results. Since the optimal seat position differs from person to person, it would be better if the subject could choose the seat position, etc., to get closer to real-world results. However, since complex instruments can interfere with radiography, a single seat position was used in this study. In addition, to minimize exposure, only one X-ray image was taken.

Third, this study included subjects between 150 and 187 cm in height, but different results may have been obtained if subjects of lesser height (for example, children) were included. In other words, the X- and Z-values may have increased in subjects shorter than 150 cm.

## Conclusions

Seated lateral radiographs of healthy volunteers were taken using an X-ray-compatible car seat, and the initial position of the lap belt was measured. The higher the BMI, the higher the X-value and Z-value, and in males, the higher the BMI, the smaller the lap belt angle. Therefore, the SBS position may be higher in obese individuals, which may increase the risk of seat belt syndrome. This analysis can help to develop safer seat belts and to enlighten car occupants.

## Data Availability

All relevant data are within the paper.
